# Adsorption and photocatalysis for methyl orange and Cd removal from wastewater using TiO_2_/sewage sludge-based activated carbon nanocomposites

**DOI:** 10.1098/rsos.170834

**Published:** 2017-12-13

**Authors:** M. Nageeb Rashed, M. A. Eltaher, A. N. A. Abdou

**Affiliations:** 1 Chemistry Department, Faculty of Science, Aswan University, 81528 Aswan, Egypt; 2 Egyptian Environmental Affairs Agency (EEAA), Aswan Branch, Aswan, Egypt

**Keywords:** photocatalyst, nanocomposite, adsorption, nanocatalyst, wastewater, heavy metals

## Abstract

Nanocomposite TiO_2_/ASS (TiO_2_ nanoparticle coated sewage sludge-based activated carbon) was synthesized by the sol-gel method. The changes in surface properties of the TiO_2_/ASS nanocomposite were characterized by X-ray diffraction (XRD), scanning electron microscope (SEM) and X-ray fluorescence. The prepared TiO_2_/ASS nanocomposite was applied for simultaneous removal of methyl orange dye (MO) and Cd^2+^ from bi-pollutant solution. The factors influencing photocatalysis (TiO_2_ : ASS ratios, initial pollutant concentrations, solution pH, nanocomposite dosage and UV irradiation time) were investigated. The results revealed that high removal efficiency of methyl orange dye (MO) and Cd^2+^ from bi-pollutant solution was achieved with TiO_2_/ASS at a ratio (1 : 2). The obtained results revealed that degradation of MO dye on the TiO_2_/ASS nanocomposite was facilitated by surface adsorption and photocatalytic processes. The coupled photocatalysis and adsorption shown by TiO_2_/ASS nanocomposite resulted in faster and higher degradation of MO as compared to MO removal by ASS adsorbent. The removal efficiency of MO by ASS adsorbent and TiO_2_/ASS (1 : 2) nanocomposite at optimum pH value 7 were 74.14 and 94.28%, respectively, while for Cd^2+^ it was more than 90%. The experimental results fitted well with the second-order kinetic reaction.

## 1. Introduction

Water pollution is a major concern all over the world. Agriculture, industry and human activities contribute to deterioration in water quality and aquatic ecosystems through the release of several pollutants such as heavy metals, dyes, surfactants, pesticides and fertilizers. Sewage sludge represents a critical environmental issue, and so the safe disposal of wastewater, sewage sludge and solid waste has become one of the major challenges to preserve the public health and the water environment [1]. Sewage sludge can be suitable as a raw material to prepare an efficient activated carbon as an adsorbent to remove pollutants such as heavy metals, colour and dyes from wastewater [2–5]. Sewage sludge, a cheap waste by-product from wastewater treatment plant, was used for the production of activated carbon. Urban sewage sludge is mainly composed of organic and inorganic substance that contains a variety of fungi and protozoa (60–70%). This organic substance is the main factor in the production of activated carbon from sewage sludge. The activated carbon can be made of residual activated sludge through high-temperature carbonization and activation, or by chemical activation.

Various techniques including photocatalysis, coagulation, chemical oxidation, adsorption and microbial degradation have been studied for dye treatment of wastewater [3,5–8]. From these techniques adsorption and photocatalysis have been widely used as effective for dye removal from wastewater. The photocatalysis is a promising advanced oxidation process, which usually uses heterogeneous titanium dioxide as a photocatalyst to degrade dyes by the decomposition and oxidation processes on its surface [3,9].

Advanced oxidation processes (AOPs) have become some of the most effective methods for the treatment of polluted water from organic pollutants, particularly low-biodegradability pollutants [10–12]. AOPs are able to complete mineralization of organic pollutants to carbon dioxide, water and inorganic compounds [13–15]. Heterogeneous photocatalysis, as one of the AOPs, is an effective method to oxidize most of the organic carbon at ambient condition [16]. The preparation of TiO_2_ coated activated carbon as a heterogeneous photocatalyst has been reported as promoting the photocatalytic efficiency of TiO_2_ and the efficiency of dye and heavy metal simultaneous removal [9,17,18]. TiO_2_ photocatalytic activity increased by increasing its surface area through the preparation of a nanostructural TiO_2_ or nanocomposite TiO_2_ with supporting materials (materials such as silica, alumina, zeolites, glass, porous nickel or clays) [19].

One of the most widely used nanocomposites for the degradation of dye-containing wastewater is TiO_2_/AC (activated carbon) composite. Several researches have been conducted using TiO_2_/AC. Xing *et al*. [6] prepared TiO_2_/AC by coatings of nanosized TiO_2_ particles on activated carbon (AC) by a sol-gel method for degradation of Rhodamine B dye. Wang *et al*. [20] prepared TiO_2_/AC composites by hydrothermal method for degradation of methyl orange dye. Jamil *et al*. [19] prepared a photocatalyst TiO_2_/AC by activated carbon impregnated with TiO_2_ for the removal of methyl orange from wastewater.

For simultaneously removing organic and inorganic pollutants from different classes, a combined substrate with a single-step process can be able to remove pollutants from different pollutants. The efficiency of the pollutant removal process from wastewaters loaded with heavy metals and dyes can be improved by using sewage sludge-based activated carbon and TiO_2_ by combining adsorption and photocatalysis techniques. So, the aims of this study are: (i) synthesis and characterization of nanocomposite TiO_2_/ASS (TiO_2_ nanoparticle coated sewage sludge-based activated carbon) with an effective TiO_2_/ASS ratio; (ii) application of the prepared nanocomposite (TiO_2_/ASS) to enhance simultaneous removal of methyl orange dye and Cd^2+^; and (iii) evaluating the effects of operational factors such as solution pH, initial pollutant concentration, nanocomposite dosage and UV irradiation time on MO and Cd removal by (TiO_2_/ASS).

## 2. Material and methods

### 2.1. Material, chemicals and reagents

Raw sewage sludge (SS) was collected from the Kima plant for sewage wastewater treatment (Aswan, Egypt). It was washed with sufficient amount of deionized water to remove dust particles and soluble matter, dried at room temperature and ground to fine powder (particle size 63 µm) by agate mortar. All chemicals and reagents used were analytically graded. The pollutants solutions were synthetically prepared using a cadmium stock solution [Cd(NO_3_)_2_ in HNO_3_ 0.5 mol l^−1^, concentration of Cd^2+^ = 1000 ± 0.002 mg l^−1^, Merck] and analytical grade of methyl orange [C_14_H_14_N_3_NaO_3_S, 99.98% purity, BDH Limited]. Titanium(IV) butoxide [Ti(OC_4_H_9_)_4,_ 97%, Aldrich].

All the batch experiments were carried out in a Pyrex conical beaker (100 ml) at room temperature under mechanical stirring (150 r.p.m.), the pH values of the sample solution were adjusted with 1N HCl or 1N NaOH, and measured by a pH meter.

**Figure 1.**
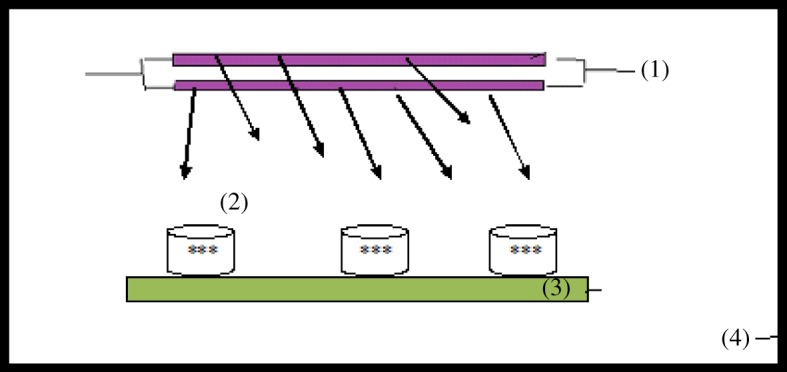
Schematic diagram of photoreactor. (1) UV irradiation lamps (power 15 watt), (2) sample solution with catalyst, (3) multi magnetic stirrer, (4) glass chamber with air conditioning.

A photoreactor consisting of multi magnetic stirrer, two UV irradiation lamps (UV-C G20/T8, *λ* 253 nm, power 15 watt), and draft chamber with air conditioning (figure 1) was used in the photodegradation process.

### 2.2. Synthesis of sewage sludge-based activated carbon (ASS)

100 g of dry sewage sludge (SS) (particle size 63 µm) was impregnated into 250 ml of 3M H_3_PO_4_ for 24 h at room temperature. After the supernatant, the liquid was removed by filtration using filter paper (Whatman 42). The precipitated sludge was dried at 105°C for 24 h, and subsequently pyrolysed at 650°C for 1 h. After cooling, the product was washed with 1M NaOH solution followed by deionized water until the pH of leached solution was between 6–7, then the resulting ASS was dried at 105°C for 24 h, crushed and sieved to <65 µm.

### 2.3. Synthesis of TiO_2_/ASS nanocomposite

Sol-gel method was applied to deposit TiO_2_ nanoparticle onto the surface of ASS. Titanium (IV) butoxide [Ti(OC_4_H_9_)_4,_ 97%, Aldrich] (50 ml) was stirred with 200 ml ethanol (HPLC grade, Fisher) for 30 min at room temperature followed by the addition of a proper amount of 1N HNO_3_ under vigorous stirring to more dispersion. When a clear transparent sol was obtained, amount of ASS was impregnated in the solution according to the preset weight ratio of TiO_2_ to ASS (1 : 3, 1 : 2 and 1 : 1). After gelation of the sol, the product was heated at 200°C in atmosphere for 2 h to obtain TiO_2_/ASS (1 : 3, 1 : 2 and 1 : 1) nanocomposite as a photocatalysts [16].

### 2.4. Materials characterization

The main chemical composition of ASS adsorbent and the nanocomposite TiO_2_/ASS (1 : 3, 1 : 2 and 1 : 1) were performed by XRF (X-ray fluorescence spectrometry; EDXRF, JOEL JSX 3222). The crystalline phases in ASS and TiO_2_/ASS (1 : 3, 1 : 2 and 1 : 1) were identified by XRD (X-ray diffraction patterns, Model: XPERT–PRO–PANalytical, The Netherlands) at the following parameters: the values of 2*θ* were in the range from 5.01° to 79.97°, Cu-K*α* radiation (*λ* = 1.54060 Å), and generator settings (30 mA, 45 kV). Surface textures were examined by SEM (scanning electron microscopy, JEOL-JSM-5500 LV).

### 2.5. Adsorption experiments by ASS (sewage sludge-based activated carbon)

MO and Cd^2+^ adsorption experiment on ASS adsorbent (100 mg/50 ml) was carried out in dark condition at room temperature with mechanical stirring (100 r.p.m.).

#### 2.5.1. MO and Cd^2+^ removal from mono-pollutant solution

100 mg of ASS adsorbent was stirred with 50 ml of mono-pollutant solution of MO (25 mg l^−1^) or Cd^2+^ (30 mg l^−1^) at solution pH 7 and contact time 5 h.

#### 2.5.2. MO and Cd^2+^ removal from bi-pollutant solution

100 mg of ASS adsorbent was stirred with 50 ml of MO and Cd^2+^ bi-pollutant solution $\left(25\;{\mathrm{mg}\;l}^{-1}\text{MO}+30\;{\mathrm{mg}\;l}^{-1}\text{C}{\text{d}}^{2+}\right)$ at solution pH 7 and contact time 5 h.

### 2.6. Photocatalytic degradation/adsorption experiments

The photocatalytic degradation experiments were carried out using photoreactor (figure 1). The nanocomposite TiO_2_/ASS (200 mg) was dumped into 50 ml of Cd^2+^ and MO bi-pollutant solution, and the UV light was turned on to initiate the photocatalytic degradation reaction for 4 h. Subsequently, the adsorption experiment of MO and Cd was carried out in the dark for 1 h to ensure the adsorption reaching an equilibrium.

### 2.7. Effect of operation factors

The effects of operation factors, such as solution pH (4, 5, 7 and 9), contact/irradiation time (0.5, 1, 2, 4, 5 and 6 h), initial pollutant concentrations [${\text{C}}_{1}(25\;{\mathrm{mg}\;l}^{-1}\text{MO}+30\;{\mathrm{mg}\;l}^{-1}\text{C}{\text{d}}^{2+})$, ${\text{C}}_{2}(100\;{\mathrm{mg}\;l}^{-1}\text{MO}+30\;{\mathrm{mg}\;l}^{-1}\text{C}{\text{d}}^{2+})$, ${\text{C}}_{3}(25\;{\mathrm{mg}\;l}^{-1}\text{MO}+100\;{\mathrm{mg}\;l}^{-1}\text{C}{\text{d}}^{2+})$ and ${\text{C}}_{4}(100\;{\mathrm{mg}\;l}^{-1}\text{MO}+100\;{\mathrm{mg}\;l}^{-1}\text{C}{\text{d}}^{2+})$] and catalyst dosage, on Cd^2+^ and MO removal by ASS adsorbent and TiO_2_/ASS (1 : 2) have been studied.

### 2.8. Analytical methods

The initial and residue concentrations of MO were measured by a double beam UV-vis spectrophotometer (Perkin Elmer135, at *λ* = 460 nm), while for Cd it was measured by atomic absorption spectrophotometer (Shimadzu, AA-6800, using air acetylene flame at *λ* = 228.8 nm). The adsorption per cent of MO and Cd^2+^ on the filter paper (0.22 µm Millipore membrane filter) and the beaker's walls were negligible (does not exceed 1%).

The removal per cent of MO and Cd^2+^ was estimated by the following equation

2.1 $$\text{Removal }\%=\frac{C_{i}-C_{r}}{C_{i}}\times 100,$$

where *C*_i_ is the initial concentration; *C*_r_ is the residue concentration at specific contact time for MO or Cd^2+^.

### 2.9. UV-vis spectra of MO solution

The UV-vis spectra of untreated MO solution (25 mg l^−1^) and treated MO solution by photocatalysts (TiO_2_/ASS) was carried out at the optimum condition to know that MO dye totally degraded or converted to intermediate compounds.

## 3. Results and discussion

### 3.1. Materials characterization

MO and Cd^2+^ removal efficiency by adsorption and photocatalysis depend on surface and structural properties of adsorbent and nanocomposite. The characterization of ASS adsorbent and TiO_2_/ASS nanocomposite were studied by X-ray fluorescence spectrometry (XRF), X-ray diffraction patterns (XRD) and scanning electron microscopy (SEM).

#### 3.1.1. X-ray fluorescence spectrometry

The chemical composition of ASS and TiO_2_/ASS (1 : 3, 1 : 2, 1 : 1) by XRF are listed in table 1.

**Table 1.** XRF chemical composition of ASS and TiO_2_/ASS (1 : 3, 1 : 2, 1 : 1).

	ms %			
element oxide	ASS	TiO_2_/ASS (1 : 3)	TiO_2_/ASS (1 : 2)	TiO_2_/ASS (1 : 1)
Al_2_O_3_	5.48	2.17	1.84	1.40
SiO_2_	18.31	7.34	5.92	4.72
P_2_O_5_	54.31	22.83	18.32	13.59
K_2_O	1.06	—	—	—
CaO	9.34	3.46	2.76	2.03
TiO_2_	1.78	55.81	64.06	72.73
ZnO	0.22	—	—	—
V_2_O_5_	—	0.75	0.90	0.99
Fe_2_O_3_	9.50	7.64	6.20	4.54

The titania TiO_2_ amount apparently increased in the nanocomposite sample with ratio TiO_2_/ASS (1 : 1). The resulting XRF chemical analysis showed the differences percentage between titania of TiO_2_/ASS with low and high contrast and titania in ASS adsorbent. TiO_2_ presented in TiO_2_/ASS nanocomposite was in higher per cent (64.06%) than in the ASS (1.78%), which confirmed the structure of TiO_2_/ASS nanocomposite.

#### 3.1.2. X-ray diffraction patterns

**Figure 2.**
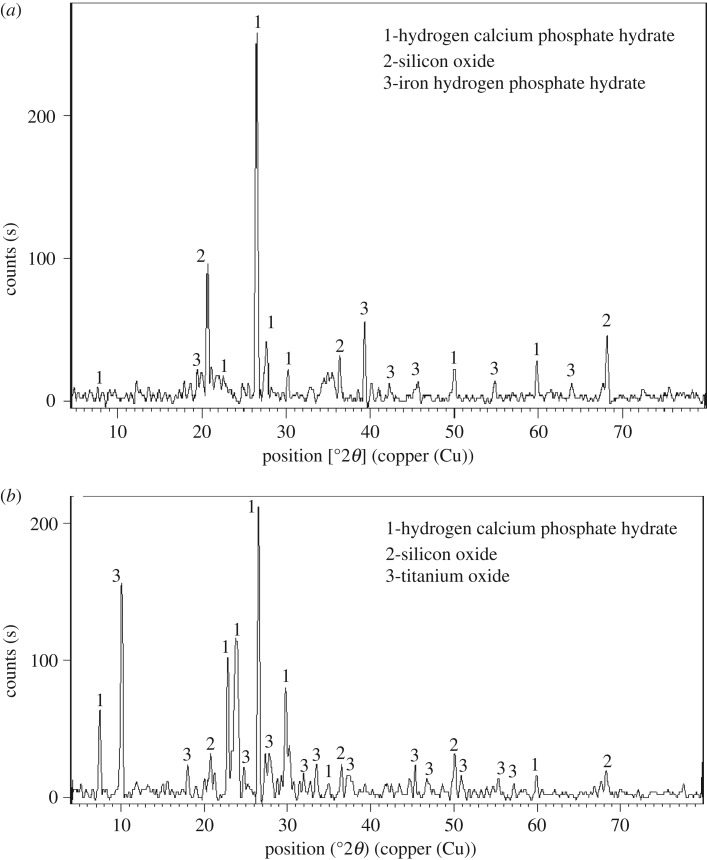
(*a*) XRD pattern of ASS adsorbent, (*b*) XRD pattern of TiO_2_/ASS (1 : 2) nanocomposite.

The crystalline phases in ASS and TiO_2_/ASS were identified by X-ray diffraction patterns at the following parameters: the values of 2*θ* were in the range from 5.01° to 79.97°, Cu-K*α* radiation (*λ* = 1.54060 Å), and generator settings (30 mA, 45 kV). The XRD data show that the major crystalline phases of ASS were silicon oxide (hexagonal), hydrogen calcium phosphate hydrate (anorthic) and iron hydrogen phosphate hydrate (figure 2). The major crystalline phases of TiO_2_/ASS are silicon oxide (monoclinic), calcium phosphate hydrate (anorthic) and titanium oxide (monoclinic). The average crystalline size of the TiO_2_ nanoparticles, calculated from the half-width of the diffraction lines in XRD pattern using the Scherrer's equation [21], was between 15.2 and 29 nm.

#### 3.1.3. Scanning electron microscopy

**Figure 3.**
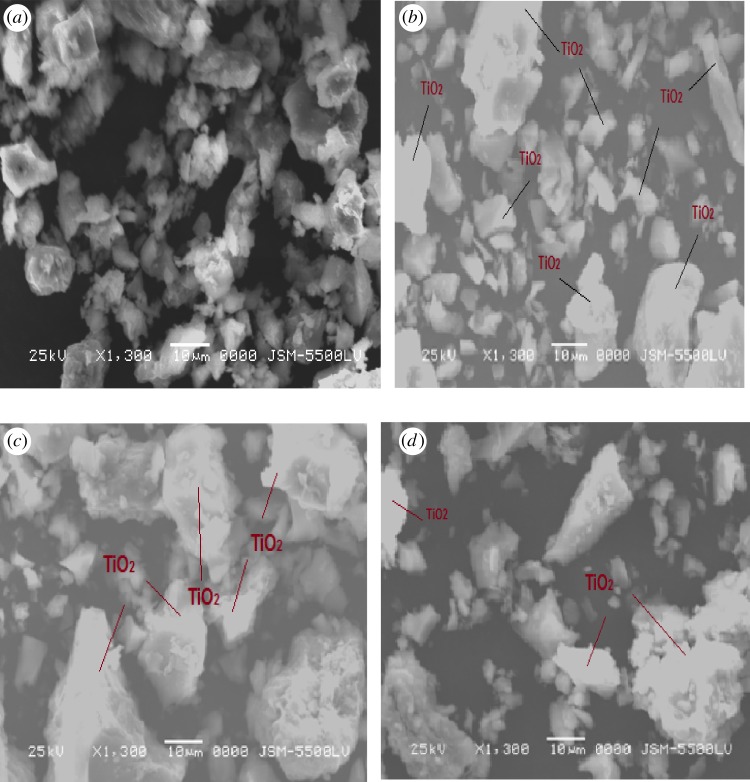
SEM images for ASS, TiO_2_/ASS (1 : 1), (1 : 2) and (1 : 3). (*a*) ASS at ×1300 and ×800. (*b*) TiO_2_/ASS (1 : 1) at ×1300 and ×850*.* (*c*) TiO_2_/ASS (1 : 2) at ×1300 and ×850 (*d*) TiO_2_/ASS (1 : 3) at ×1300 and ×1600.

The SEM was investigated by studying the surface morphology of the ASS adsorbent and TiO_2_/ASS (1 : 3, 1 : 2, 1 : 1) nanocomposite (figure 3). The ASS and TiO_2_*/*ASS materials showed different aspects at relatively low magnification; the ASS appeared smooth, while the TiO_2_*/*ASS material was rough. Moreover, the different rates of TiO_2_ uniformly dispersed on ASS surface in coated ASS according to TiO_2_. High amount of titanium particles was deleted for TiO_2_/ASS nanocomposite. It would be expected that with the increase of TiO_2_ dispersion rate on ASS surface the photocatalytic activity of catalyst would be more powerful.

### 3.2. Optimal TiO_2_/ASS nanocomposite ratio selection

**Figure 4.**
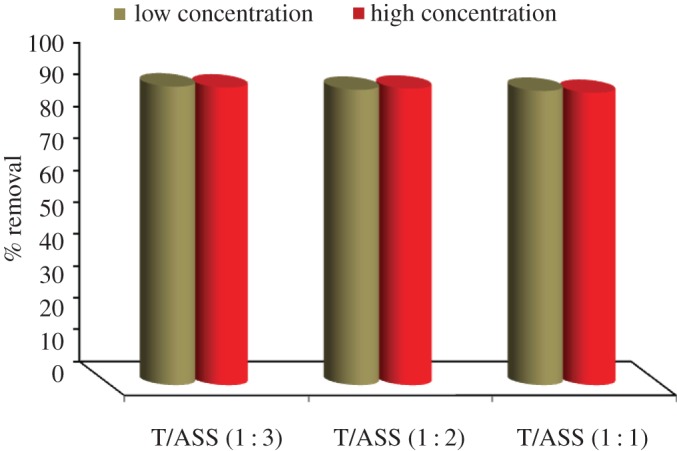
Removal efficiency of MO by TiO_2_/ASS (1 : 3, 1 : 2 and 1 : 1) at low and high MO and Cd^2+^ initial concentration.

**Figure 5.**
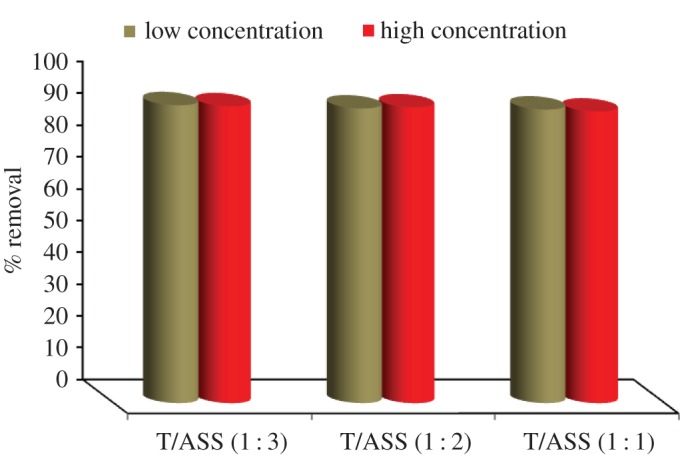
Removal efficiency of Cd^2+^ by TiO_2_/ASS (1 : 3, 1 : 2 and 1 : 1) at low and high MO and Cd^2^^+^ initial concentration.

The optimal effective ratio of TiO_2_/ASS (1 : 3, 1 : 2 and 1 : 1) nanocomposite for MO degradation and Cd^2+^ adsorption was studied. Figure 4 shows that the photocatalytic activity of TiO_2_/ASS (1 : 3, 1 : 2 and 1 : 1) for MO dye removal is in the following order: TiO_2_/ASS (1 : 2) > TiO_2_/ASS (1 : 1) > TiO_2_/ASS (1 : 3). The removal efficiency of Cd^2+^ adsorption by TiO_2_/ASS (1 : 3, 1 : 2 and 1 : 1) ranging from 93.67 to 93.33%, from 92.67 to 92.00% and from 92.33 to 91.67%, respectively (figure 5), while the order of Cd^2+^ adsorption efficiency is TiO_2_/ASS (1 : 3) > TiO_2_/ASS (1 : 2) > TiO_2_/ASS (1 : 1).

The results indicate that the most effective weight ratio of TiO_2_ to ASS is (1 : 2), which is suitable for high efficiency for MO degradation and Cd^2+^ adsorption from bi-pollutant solution. So, in the rest of the experiments TiO_2_/ASS (1 : 2) will be used.

Jamil & Sharaf El-Deen [22] prepared TiO_2_ nanoparticles on calcined sewage sludge (TiO_2_/sludge). The photocatalytic efficiency of TiO_2_/sludge was evaluated by tartrazine dye degradation by halide lamp. TiO_2_/sludge exhibited a high photocatalytic oxidation efficiency (more than 90%) of tartrazine compared with naked TiO_2_ (less than 20%) due to the synergy effect of sewage sludge.

Several researches prepared activated carbon in TiO_2_/activated carbon composite from different raw materials and applied it for the removal of methyl orange dye and Cd. Sharaf El-Deen & Zhang [23] prepared TiO_2_/sewage sludge (TS) as biomass material and found that the adsorption removal of Cd was 74%. Modified fly ash was used for the preparation of TiO_2_/activated carbon and used for the removal of 82% Cd from polluted solution [18]. Visa & Duta [24] applied modified fly ash (FA) mixed with TiO_2_ photocatalyst for simultaneous removal of methyl orange and cadmium from polluted water. The removal of MO and Cd by TiO_2_/ASS photocatalyst was 94.28 and 93.67%, respectively. These results were less than our finding. Carbon foam-loaded nano-TiO_2_ photocatalyst was prepared and used for photodegradation of MO dye with removal per cent by 83–87% [25]. Carbonized cotton T-shirt loaded nano-TiO_2_ photocatalyst was examined by the degradation of methyl orange up to 98.6% [26]. These results were near our finding.

### 3.3. Adsorption and photocatalytic activity

In this study a nanocomposite TiO_2_/ASS and ASS adsorbent were applied for simultaneous removal of MO dye and Cd^2+^ from bi-pollutant solutions. The application proceeded through two systems: adsorption on ASS adsorbent in darkness, and photocatalysis by nanocomposite TiO_2_/ASS under UV irradiation.

#### 3.3.1. Adsorption system (ASS) in dark

**Figure 6.**
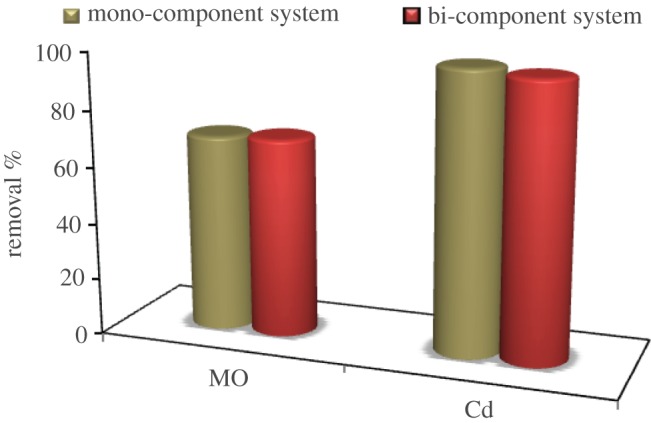
Removal efficiency of MO and Cd^2+^ by ASS adsorbent from mono- and bi-component pollutants.

Figure 6 shows that MO dye and Cd^2+^ removal efficiency by ASS adsorbent from mono-pollutant solution was 69.8 and 98.13%, respectively, while from bi-pollutant solution it was 70.28 and 96.7%, respectively. These results indicate that Cd^2+^ adsorption efficiency is more than MO adsorption efficiency in both mono- and bi-pollutant solutions. A competition is expected on the active sites of ASS between Cd^2+^ and MO in bi-pollutant solution, but MO dye adsorption on ASS surface is less favoured as a result of the ionized form of MO dye having a negatively charged head.

It suggested that MO and Cd^2+^ adsorption is processed as follows: (i) Cd^2+^ adsorbed on the ASS surface by attraction force and chemical binding; (ii) MO dye adsorbed on available active site on the heterogeneous ASS surface and the interaction with cadmium cations where the amine head in methyl orange structure can act as electron donors according to Visa & Duta [24].

#### 3.3.2. Adsorption/photocatalysis system (TiO_2_/ASS)

**Figure 7.**
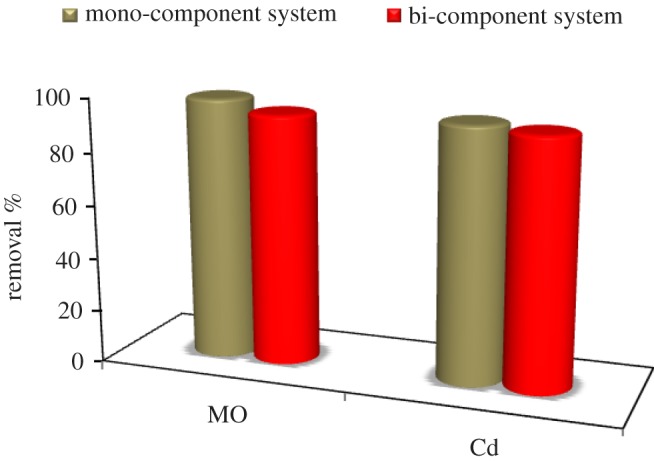
MO and Cd^2+^ removal efficiency from mono- and bi-component pollutant solution by TiO_2_/ASS (1 : 2) nanocomposite*.*

The data given in figure 7 show that MO and Cd^2+^ removal efficiency by TiO_2_/ASS (1 : 2) in mono-pollutant solution was 99 and 95%, respectively, while in bi-pollutants solution it was 94.92 and 92.97%, respectively. This result indicates that MO and Cd^2+^ removal efficiency by TiO_2_/ASS (1 : 2) in bi-pollutants solution decreases from 99 to 94.92 and from 95 to 92.97, respectively, compared to that in mono-pollutant solution. This may be due to the expected competition between MO and Cd^2+^ on the active sites of TiO_2_/ASS (1 : 2).

Silica (SiO_2_) as a part of sewage sludge activated carbon acts as an effective adsorbent site on the surface of TiO_2_/ASS for the adsorption of MO dye and Cd. The developed new active sites (SiO^−^) on TiO_2_/ASS surface, allow Cd^2+^ to form complexes on the surface [27] as described below:

3.1 $$2(\equiv \text{Si}{\text{O}}^{-})+\text{C}{\text{d}}^{2+}\to {\left(\equiv \text{Si-O}\right)}_{2}\text{Cd}.$$

The TiO_2_-based compounds on the TiO_2_/ASS surface are expected to host similar processes. On the TiO_2_/ASS, simultaneous processes of adsorption and photocatalysis of MO and Cd will be developed, according to equation (3.2):
3.2
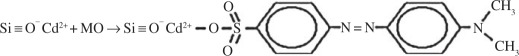


### 3.4. Integration of adsorption and photocatalytic degradation of methyl orange using TiO_2_/ASS nanocomposite

**Figure 8.**
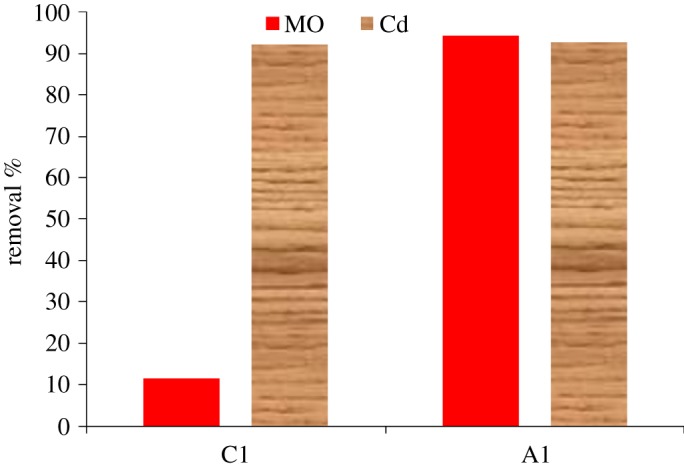
Integration of adsorption and photocatalytic degradation of methyl orange using TiO_2_/ASS nanocomposite (C1, Adsorption; A1 Photocatalysis).

The experiments were carried out in two consecutive conditions: the first one (C1) included adsorption of MO and Cd using TiO_2_/ASS nanocomposite in a dark condition for 1 h, while the second one (A1) was conducted under UV irradiation (UV-C G20/T8, *λ* = 253 nm) for 4 h. Experimental conditions were fixed at pH 7 and nanocomposite dose 200 mg. The data given in figure 8 show that the MO removal efficiency by A1 and C1 conditions was 94.28 and 11.6%, respectively. These results indicate that most of MO concentration was removed by photocatalytic degradation mechanism and not by adsorption mechanism.

### 3.5. Effect of operation factors

#### 3.5.1. Effect of solution pH

**Figure 9.**
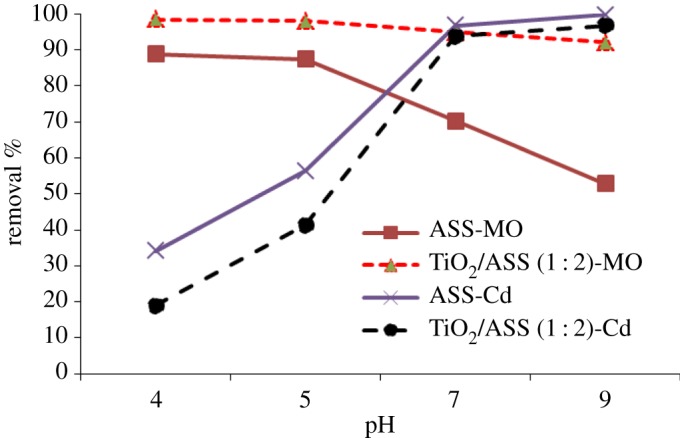
Effect of solution pH on MO and Cd^2+^ removal efficiency by ASS or TiO_2_/ASS (1 : 2).

The effects of solution pH on MO and Cd^2+^ removal efficiencies were investigated at pH values 4, 5, 7 and 9, with constant conditions of initial concentration $\left(25\;{\mathrm{mg}\;l}^{-1}\text{MO}+30\;{\mathrm{mg}\;l}^{-1}\text{C}{\text{d}}^{2+}\right)$, and 5 h contact time. The results in figure 9 show that as solution pH increases from 4 to 9, the MO dye removal efficiency by ASS and TiO_2_/ASS (1 : 2), decreases from 88.8 to 52.8% and from 98.4 to 92%, respectively. The previous results indicated that at pH values ranging between 4 and 5 the MO removal by ASS and TiO_2_/ASS (1 : 2) was effective. These results are consistent with reports that photocatalysis process can remove pollutants under both acidic and neutral conditions [28,29]. The MO removal by ASS adsorbent was more effective at acidic range (pH 4–5), where at low pH values more protons were available causing an increase in electrostatic attraction between negatively charged MO dye anions and positive charge on ASS surface and this resulted in an increase in MO adsorption capacity. At basic medium the positive charge on the ASS surface decreased and repulsion between anionic dye molecules and the excessive hydroxide ions resulted in a sharp decrease in adsorption, and so the acidic range is the most appropriate for MO removal [30].

Cd^2+^ removal efficiency by ASS and TiO_2_/ASS (1 : 2) nanocomposite clearly increases with increasing pH value from 4 to 9 to reach maximum removal of Cd at 99.6%, and 98.3%, respectively at pH 9. This is due to that at an acidic medium more H^+^ ions were available, which led to repulsion between Cd^2+^ and active sites on the surface of substrate. So, subsequently a decrease in Cd^2+^ adsorption was observed, whereas with the increase of pH value the ASS surface is negatively charged and attracts positive cadmium ions (Cd^2+^). At pH < 8 Cd^2+^ removal depends on the adsorption mechanism only, while at pH > 8 cadmium precipitation occurs with adsorption where cadmium ions form hydroxides Cd(OH)^+^ or Cd(OH)_2_ [31,32].

#### 3.5.2. Effect of MO and Cd^2+^ initial concentration

**Figure 10.**
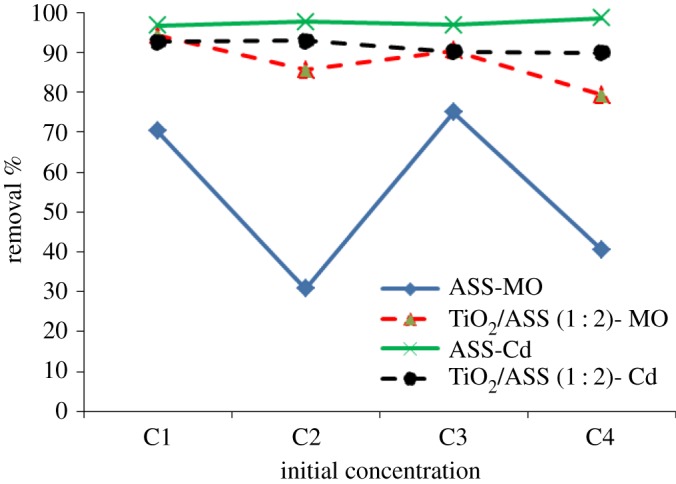
Effect of MO and Cd^2^^+^ initial concentration on MO and Cd^2+^ removal efficiency by ASS or TiO_2_/ASS (1 : 2).

Effect of MO and Cd^2+^ initial concentration on its removal efficiency was studied at initial concentrations [${\text{C}}_{1}(25\;{\mathrm{mg}\;l}^{-1}\text{MO}+30\;{\mathrm{mg}\;l}^{-1}\text{C}{\text{d}}^{2+})$, ${\text{C}}_{2}(100\;{\mathrm{mg}\;l}^{-1}\text{MO}+30\;{\mathrm{mg}\;l}^{-1}\text{C}{\text{d}}^{2+})$, ${\text{C}}_{3}(25\;{\mathrm{mg}\;l}^{-1}\text{MO}+100\;{\mathrm{mg}\;l}^{-1}\text{C}{\text{d}}^{2+})$ and ${\text{C}}_{4}(100\;{\mathrm{mg}\;l}^{-1}\text{MO}+100\;{\mathrm{mg}\;l}^{-1}\text{C}{\text{d}}^{2+})$], with constant conditions of pH 7, and 5 h contact time. The data in figure 10 indicate that TiO_2_/ASS (1 : 2) nanocomposite showed higher MO removal efficiency than that by ASS using initial pollutant concentrations (C_1_, C_2_, C_3_ and C_4_). The Cd^2+^ removal efficiency by ASS adsorbent or TiO_2_/ASS (1 : 2) nanocomposite was ≥90%, while the removal efficiency of Cd^2+^ and MO by ASS adsorbent decrease with increasing Cd^2+^ and MO initial concentrations due to that at high initial concentrations the ratio between Cd^2+^ and MO initial concentration to the number of available adsorption sites on ASS surface was high which led to decrease in adsorption rate [31]. In photocatalysis with TiO_2_/ASS (1 : 2) nanocomposite the increase in MO dye concentration leads to reduction of UV radiated on the active sites of catalyst and low OH^•^ radical production where the active sites may be occupied by dye ions and intermediate products formed during dye oxidation [33–35].

#### 3.5.3. Effect of contact (or irradiation) time

**Figure 11.**
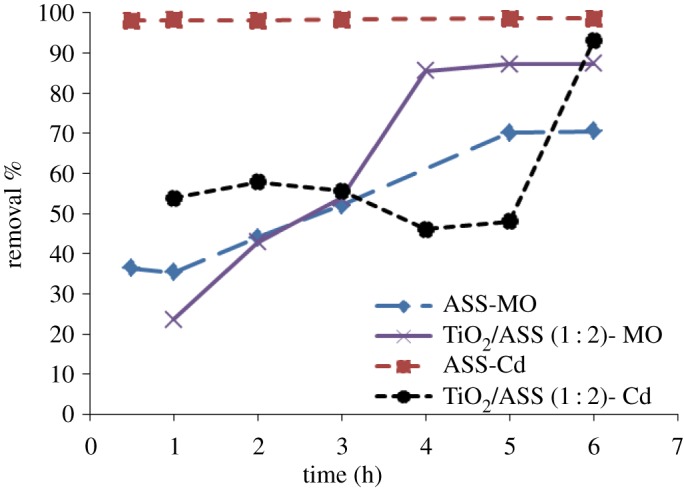
Effect of contact time on MO and Cd^2^^+^ removal efficiency by ASS or TiO_2_/ASS (1 : 2).

To determine Cd^2+^ and MO removal rate by ASS and TiO_2_/ASS (1 : 2), the effect of contact (or irradiation) was investigated at various time values ranging from 30 to 360 min, with constant pH 7, and initial concentration $\left(25\;{\mathrm{mg}\;l}^{-1}\text{MO}+30\;{\mathrm{mg}\;l}^{-1}\text{C}{\text{d}}^{2+}\right)$. The results presented in figure 11 show that the MO removal rate by ASS and TiO_2_/ASS (1 : 2) increased with increasing time and almost reached a plateau after approximately 300 min. The Cd^2+^ removal rate by ASS was very rapid during the first 30 min where Cd^2+^ removal efficiency reached to 98.13%. Cd^2+^ removal rate by TiO_2_/ASS (1 : 2) was rapid during the first 30 min, then it continued at a slower rate during the time between 30 to 300 min and it was rapid in the last 60 min. In the adsorption process rapid MO and Cd^2+^ adsorption rate was observed during the first 30 min, due to large numbers of free adsorption sites being available for MO and Cd^2+^ adsorption, whereas the slow absorption rate was observed due to lesser number of active adsorption sites and a competition between MO and Cd^2+^ expected on the active sites. In TiO_2_/ASS (1 : 2) with the increase of irradiation time the MO degradation rate increased and more active sites for Cd^2+^ adsorption were available [24,36].

### 3.6. UV-vis spectra of MO solution

**Figure 12.**
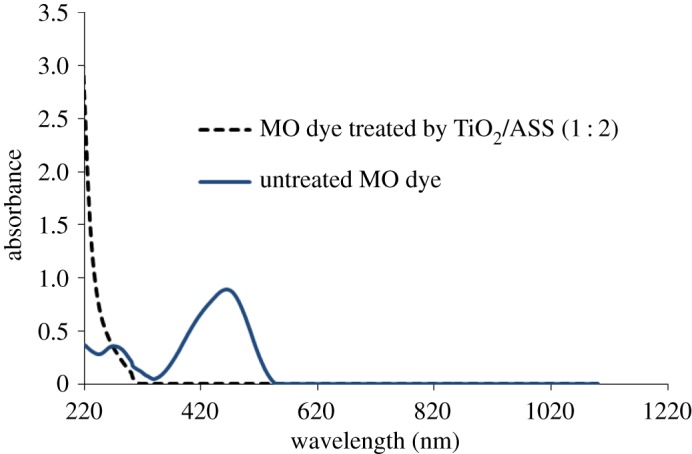
UV-vis spectra of untreated MO solution and treated MO solution by TiO_2_/ASS (1 : 2).

UV-vis spectra of untreated MO solution and treated MO solution by TiO_2_/ASS (1 : 2) was carried out at the optimum condition. From figure 12 it is clear that untreated MO solution showed two absorption peaks at 460 and 290 nm corresponding to azo band (–N=N–) and benzene rings in MO molecule [37], whereas the two peaks at 460 and 290 nm completely disappeared with a treated MO solution by TiO_2_/ASS (1 : 2).

### 3.7. Kinetic models

Kinetics of MO and Cd^2+^ simultaneous removal by ASS and TiO_2_/ASS (1 : 2) were analysed using pseudo-first-order and pseudo-second-order kinetic models [38,39].

Pseudo first order is expressed by the following equation:

3.3 $$\text{log }(q_{e}-q_{t})=\text{log }q_{e}-\frac{k_{f}}{2.303}t,$$

where *q*_e_ (mg gm^−1^) and *q_t_* (mg gm^−1^) are the amounts of sorbates adsorbed on the sorbents at equilibrium and at time *t*, respectively; *k*_f_ (min^−1^) is the rate constant of pseudo-first-order kinetic model and *t* (min) is the agitation time. The kinetic parameter *k*_f_ and correlation coefficient can be obtained from the plot of log (*q*_e_ − *q_t_*) versus *t*.

Pseudo second order is expressed by the following equation:

3.4 $$\frac{t}{q_{t}}=\frac{1}{k_{s}.{q_{e}}^{2}}+\frac{t}{q_{e}},$$

where the pseudo-second-order kinetic constant represented as *k*_s_ (gm mg^−1^ min^−1^). The kinetic parameters of the experimental data can be determined by plotting *t*/*q_t_* against *t*.

Based on the values of correlation coefficient (*r*^2^), theoretical and experimental values of *q*_e,_ the MO and Cd^2+^ removal rates by ASS and TiO_2_/ASS (1 : 2), followed pseudo-second-order model (table 2). The values of *k*_s_ refer to that the MO and Cd^2+^ removal rate by ASS was faster than that by TiO_2_/ASS (1 : 2); moreover, Cd^2+^ removal rate was faster than MO removal rate by ASS and TiO_2_/ASS (1 : 2). The *q*_e(s)_ values of Cd^2+^ removal by ASS, and MO removal by ASS system were 14.79, and 8.89 mg g^−1^, respectively, which were very close to the experimental data (14.7 and 7.87 mg g^−1^, respectively).

**Table 2.** Kinetic parameters for Cd^2+^ and MO removal by ASS and TiO_2_/ASS (1 : 2).

	pseudo first order			pseudo second order			
system	*q*_e(f)_	*k_f_*	*r*^2^	*q*_e(s)_	*k*_s_	*r*^2^	*Q*_exp_.
MO – ASS	4.48	0.006	0.920	8.896	0.002	0.969	7.87
MO – TiO_2_/ASS (1 : 2)	59.25	0.023	0.916	30.12	0.0002	0.981	21.83
Cd^2+^ – ASS	12.33	0.004	0.645	14.79	0.205	1	14.78
Cd^2+^ – TiO_2_/ASS (1 : 2)	2.65	0.001	0.7156	3.42	0.026	0.995	6.97

### 3.8. MO and Cd^2+^ adsorption isotherms

Langmuir and Freundlich isotherm models show the relationship between MO and Cd^2+^ concentration in solution and the adsorbed amount of MO and Cd^2+^ at a constant temperature. Langmuir adsorption model describes monolayer adsorption which occurs at homogeneous sites of the outer surface of adsorbent. The linear form of Langmuir isotherm is given by the following equation:

3.5 $$\frac{1}{q_{e}}=\frac{1}{Q_{o}}+\frac{1}{bQ_{o}C_{e}},$$

where *C*_e_ is the equilibrium concentration of the adsorbate (mg l^−1^), *q*_e_ is the amount adsorbed (mg g^−1^), and *Q*_o_ and *b* are the Langmuir constants related to maximum adsorption capacity and energy of adsorption, respectively. When *C*_e_/*q*_e_ is plotted versus *C*_e_, the slope is equal to (1/*Q*_o_) and the intercept is equal to 1/*Q*_o_*b*.

Freundlich adsorption model assumes heterogeneous adsorption due to the diversity of adsorption sites. This isotherm can be described as:

3.6 $$\text{log}\;q_{e}=\text{log}\;k_{f}+\left(\frac{1}{n}\right)\text{log}\;C_{e},$$

where *k*_F_ is Freundlich constant, 1/*n* is adsorption intensity, When log *q*_e_ is plotted versus log *C*_e_, the slope is equal to (1/*n*) and the intercept is equal to log *k*_f_. The data from table 3 indicate that Cd^2+^ adsorption by ASS and TiO_2_/ASS (1 : 2) is fitting to the Freundlich model. The values of 1/*n* were between 0 to 1 which refer to the heterogeneity of the ASS and TiO_2_/ASS (1 : 2) [40]; furthermore, the values of *k*_f_ indicate that ASS sorbent has higher adsorption capacity and affinity for Cd^2+^ than TiO_2_/ASS (1 : 2) [41]. The MO adsorption by ASS fitted well to the Langmuir model and the MO maximum adsorption by ASS is 16.61 mg g^−1^.

**Table 3.** Adsorption isotherm parameters for Cd^2+^ and MO adsorption.

	Langmuir			Freundlich		
system	*Q*_o_	*b*	*r*^2^	1/*n*	*K*_f_	*r*^2^
MO – ASS	16.61	0.17	0.999	0.22	5.9	0.97
Cd^2+^ – ASS	212.7	0.10	0.082	0.911	20.6	0.706
Cd^2+^ – TiO_2_/ASS (1 : 2)	58.4	0.06	0.992	0.881	3.87	0.999

## 4. Conclusion

Nanocomposite TiO_2_/ASS (TiO_2_ nanoparticle coated sewage sludge-based activated carbon) was prepared successfully and characterized. TiO_2_/ASS (1 : 2) nanocomposite showed high efficiency for treatment of wastewater containing mixture of dye (MO) and heavy metal (Cd). The application of photocatalysis/adsorption leads to maximize MO and Cd^2+^ simultaneous removal efficiency compared to adsorption processes. Cd^2+^ removal efficiency by ASS adsorbent or TiO_2_/ASS (1 : 2) nanocomposite was ≥90% at optimum condition. Solution pH, contact (or irradiation) time, adsorbent and nanocomposite dosage showed a direct effect on the MO and Cd removal efficiencies. The data of MO and Cd^2+^ removal fitted very well to pseudo-second-order model, while MO removal rate was slower than Cd^2+^ removal rate during photocatalysis.

## Permission to carry out fieldwork

The study does not contain fieldwork.

## Data accessibility

All of the data in this investigation have been reported in the paper and are freely available.
